# Letter to the Editor regarding ‘Unique pharmacodynamic properties and low abuse liability of the µ-opioid receptor ligand (S)-methadone’

**DOI:** 10.1038/s41380-024-02621-6

**Published:** 2024-05-28

**Authors:** Marco Pappagallo, Thomas R. Kosten, Charles W. Gorodetzky, Frank J. Vocci, Frank L. Sapienza, Sara De Martin, Stefano Comai, Andrea Mattarei, Charles E. Inturrisi, Paolo L. Manfredi

**Affiliations:** 1 Relmada Therapeutics, Inc., Coral Gables, FL USA; 2 Baylor College of Medicine, MD Anderson Cancer Center, University of Houston, Houston, TX USA; 3 Consultant in Pharmaceutical Medicine, Kansas City, MO USA; 4 Friends Research Institute, Baltimore, MD USA; 5 The Drug and Chemical Advisory Group LLC, Fairfax, VA USA; 6 Department of Pharmaceutical and Pharmacological Sciences, University of Padua, Padua, Italy; 7 Department of Pharmaceutical and Pharmacological Sciences, and Department of Biomedical Sciences, University of Padua, Padua, Italy; 8 Department of Psychiatry, McGill University, Montreal, QC Canada

**Keywords:** Neuroscience, Drug discovery

## To the editor:

We wish to commend Levinstein and colleagues for their contribution to our understanding of the pharmacodynamic (PD) properties of esmethadone [(S)-MTD] at mu opioid receptors (MORs) and at N-methyl-D-aspartate receptors (NMDARs) [1]. We agree with their conclusion that (S)-MTD could potentially become a safe and effective drug for a multiplicity of disorders. However, in the Discussion section, the authors state: “Thus, the currently assumed role of NMDAR blockade in the purported antidepressant effects of (S)-MTD should be reassessed and explained in the frame of its MOR agoniztic properties”.

The critical experiment supporting Levinstein’s explanation of MOR agonism for the antidepressant effect of (S)-MTD is the demonstration of hot plate analgesia induced by both levomethadone [(R)-MTD] and (S)-MTD, in contrast to the results of previous studies, showing no hot plate analgesia with (S)-MTD [2–4]. This discrepancy appears due to the high dose used by Levinstein et al., which was 35-fold higher for (S)-MTD than for (R)-MTD. These high doses were justified by similar (S)-MTD doses used in models of depressive like behavior [1]. The 17.9 mg/kg rat dose of (S)-MTD required for hot plate analgesia is equivalent to a human dose of approximately 200 mg (S)-MTD [5], which is above the maximum human tolerated dose of 150 mg (S)-MTD [6].

In contrast to their inference of (S)-MTD meaningful opioid agonist effects based on hot plate analgesia at high doses, the authors show specific loss of efficacy for (S)-MTD at the MOR-Gal1R heteromer. (S)-MTD did not produce increases in extracellular dopamine and counteracted dopamine release induced by (R)-MTD. The authors also show that (S)-MTD does not exert full cataleptic effects, does not increase locomotor activity and does not elicit self-administration, unlike full opioid agonizts. The self-administration of (S)-MTD seen by Levinstein following discontinuation of (R)-MTD [1] may indicate NMDAR antagonism mitigating opioid withdrawal caused by (R)-MTD discontinuation [7], rather than suggesting opioid agonist effects. In summary, except for the noted analgesic effects at high doses, which could also be explained by NMDAR antagonistic activity [4], the findings presented by Levinstein et al., are consistent with prior preclinical studies indicating that (S)-MTD has no meaningful opioid-like agonist effects [2–4] and no meaningful abuse potential [8]. The general consensus on the lack of meaningful opioid agonist effects by (S)-MTD is also consistent with recent clinical studies showing no signal of meaningful abuse potential [9, 10] and no indication of meaningful opioid agonist-like effects [4, 11, 12]. Consistent with this lack of opioid-like reinforcing effects of (S)-MTD in animal and in human studies, Levinstein shows that (S)-MTD can antagonize the effects of (R)-MTD [1]. Notably, the lack of meaningful MOR agonism in humans is confirmed by recent clinical studies where (S)-MTD was administered to hundreds of subjects without signals for the typical euphoric, respiratory depressant, constipating and withdrawal effects typical of opioid agonist drugs [4, 9–12].

The authors exclude meaningful NMDAR activity of (S)-MTD based on their noted lack of NMDAR occupancy at potentially relevant concentrations. A current hypothesis for the MOA of (S)-MTD as an uncompetitive NMDAR antagonist antidepressant consists in the preferential block of a small percentage of GluN2D subtypes which are pathologically hyperactive at resting membrane potential in neurons part of circuits relevant for patients with MDD [13]. Blockade of these NMDARs results in suppression of Ca^2+^ signals at resting membrane potential with dephosphorylation of eEF2 leading to de-suppression of dendritic protein synthesis [14]. The blockade of NMDA receptor at resting membrane potential remains a primary hypothesis for the antidepressant MOA for uncompetitive NMDAR antagonists [15, 16]. The drug exposure resulting from (S)-MTD doses used in MDD clinical trials is compatible with a hypothesized antidepressant MOA that pivots on the activity-dependent block of a small percentage of NMDARs at graded resting membrane potential in neurons part of circuits relevant for MDD [13]. Levinstein’s procedures would not be expected to show detectable NMDAR occupancy because the activity-dependent NMDAR open channel block by (S)-MTD is limited to the small percentage of pathologically hyperactive NMDARs at resting membrane potential [1].

Pharmacodynamic studies of NMDAR antagonists need to address not only occupancy and displacement affinity, but also measurements of Ca^2+^ fluxes by different modalities [17], performed in the absence and in presence of Mg^2+^ [17, 18] and should also measure and account for the degree of “trapping” within the NMDAR channel pore [17, 19]. Additionally (S)-MTD is a positively charged molecule at physiological pH, a feature that may contribute to its activity-dependent NMDAR block [13, 20]. Affinity, activity, trapping, and protonation are all important determinants of the relative potency and subtype preference of activity-dependent uncompetitive NMDAR antagonists. Current hypothesized MOA for (S)-MTD and other uncompetitive NMDAR antagonists require blocking only a small percentage of pathologically hyperactive and Mg^2+^ free NMDARs, which are open at resting membrane potential and are expressed at the membrane of neurons part of select circuits relevant to patients with MDD [13–16]. NMDARs in the closed configuration and physiologically blocked by Mg^2+^ are not susceptible to (S)-MTD block. (S)-MTD and other protonated low potency NMDAR antagonists are unlikely to engage NMDARs at action potential. Furthermore, clinically tolerated uncompetitive NMDAR antagonists are not required to cause psychopathological dissociation in order to exert antidepressant effects [11, 12, 21, 22], supporting the notion that only limited NMDAR occupancy is required for antidepressant therapeutic effects. The degree of NMDAR occupancy by (S)-MTD required to exert antidepressant activity in humans may therefore be below the threshold of the experimental measures used by Levinstein and colleagues [1].

Levinstein’s experiments add to our understanding of the PD of (S)-MTD, (R)-MTD and (R,S)-MTD [1] but do not support an opioid agonist antidepressant mechanism and do not exclude activity-dependent block of pathologically hyperactive NMDARs at resting membrane potential as the antidepressant mechanism for (S)-MTD.

In summary, the understanding of the precise mechanisms by which (S)-MTD and other NMDAR antagonists exert antidepressant effects remains a fascinating work in progress with the potential for advancing our understanding of CNS pathophysiology. In the meantime, blocking NMDARs at resting membrane potential remains a well-grounded hypothesis for explaining the antidepressant effects of ketamine and other NMDAR antagonists [14–16], including (S)-MTD [13].
